# Preface

**DOI:** 10.1098/rstb.2021.0491

**Published:** 2022-03-14

**Authors:** Marina Rafajlović, Jake M. Alexander, Roger K. Butlin, Kerstin Johannesson

**Affiliations:** ^1^ Department of Marine Sciences, University of Gothenburg, 405 30 Gothenburg, Sweden; ^2^ Centre for Marine Evolutionary Biology, University of Gothenburg, 405 30 Gothenburg, Sweden; ^3^ Department of Environmental Systems Science, ETH Zürich, 8092 Zürich, Switzerland; ^4^ Ecology and Evolutionary Biology, School of Biosciences, University of Sheffield, Sheffield S10 2TN, UK; ^5^ Department of Marine Sciences, University of Gothenburg, Tjärnö Marine Laboratory, 452 96 Strömstad, Sweden

Species’ distributions change over time. Although in a given time span a species' range may seem static, it may have undergone major changes in the past and it is likely to change in the future. For example, ranges can change in size by expanding or contracting, or shift in space while either preserving or changing their size. Furthermore, ranges can exhibit changes in the extent of (local) fragmentation, and this may or may not occur jointly with changes in range size and/or shifts. These changes can be driven by modifications to environmental factors that limit ranges, which may be biotic or abiotic, or by evolutionary changes within species, or both. Importantly, a change in a species’ range may impact on populations, species, communities or ecosystem functioning in the newly occupied or abandoned habitats, with the invasion of non-native species often expected to induce negative effects. Understanding the dynamics of species' ranges thus provides the core for understanding the dynamics of biodiversity. This is a central task of modern biology, of great relevance to society, not least owing to ongoing global climate change, which has already caused range alterations of many species and loss of biodiversity, with more changes expected.

This theme issue synthesizes existing knowledge and hypotheses on the modification of species’ ranges, and extends this knowledge with new hypotheses, empirical and theoretical results. Topics addressed include the role of the environmental conditions, including biotic and abiotic factors, that the species has encountered in the past and is encountering in the present, and the role of species-specific intrinsic properties, including dispersal ability of individuals, intrinsic growth rate, niche requirements, plasticity and adaptive potential for niche evolution, among others.

We hope that the theme issue will contribute to answering questions of the type: How will a range change (if at all) given a scenario of interest, at what rate, and with what consequences? Answering this sort of question is essential to design successful conservation and management actions for conserving biodiversity or mitigating its loss.

This theme issue consists of two parts. Part I begins with a discussion of macroecological patterns in species' ranges and provides a new hypothesis for how these patterns might themselves be generated through range evolution [1]. Three contributions then consider the role of plasticity in influencing range limits [2–4], followed by consideration of effects at range margins of pollen limitation [5] and genetic load [6]. Range expansion can cause surfing of underdominant alleles [7]. Finally, three contributions move towards management considerations, starting with biophysical modelling of dispersal [8] and reconstruction of invasion history [9] and then considering management of human-mediated range change [10].

Part II will include further contributed papers, a general Introduction [11] and a Conclusion that draws together all contributions to the theme issue [12].

## Data accessibility

This article has no additional data.
